# Effects of an AI-enhanced BOPPPS teaching model in nursing courses: a meta-analysis of randomized controlled trials

**DOI:** 10.3389/fmed.2026.1767911

**Published:** 2026-02-18

**Authors:** Huiling Zhang, Zheyuan Xia, Yahui Meng, Shuang Yu, Hui Shi

**Affiliations:** Laboratory of Geriatric Nursing and Health, Anhui University of Traditional Chinese Medicine, Hefei, China

**Keywords:** AI, BOPPS, education, meta, nursing

## Abstract

**Aim:**

To evaluate the effectiveness of an artificial intelligence (AI)–enhanced BOPPPS teaching model in nursing courses, with a focus on academic performance, self-directed learning ability, and teaching satisfaction.

**Design:**

Systematic review and meta-analysis of randomized controlled trials (RCTs) conducted in accordance with PRISMA and registered in INPLASY (INPLASY202590123).

**Methods:**

Chinese- and English-language databases were searched for eligible RCTs comparing AI-enhanced BOPPPS with traditional teaching (or BOPPPS alone). Data extraction and risk-of-bias assessment followed Cochrane guidance. Pooled analyses were performed using standardized mean difference (SMD) for continuous outcomes and risk ratio (RR) for dichotomous outcomes, with heterogeneity assessed using I^2^.

**Results:**

Four RCTs involving 459 nursing students were included. Academic performance showed a significant improvement in the AI-enhanced BOPPPS group (SMD = 1.06, 95% CI 0.63–1.48; *p* < 0.05), with substantial heterogeneity. With substantial heterogeneity. Self-directed learning ability (3 studies; “excellent” category) was significantly improved in the AI-enhanced BOPPPS group (RR = 3.28, 95% CI 2.14–5.02; *p* < 0.001) with no heterogeneity. Teaching satisfaction was also significantly higher with AI-enhanced BOPPPS (RR = 1.80, 95% CI 1.27–2.55; *p* < 0.001), with low-to-moderate heterogeneity.

**Conclusion:**

AI-enhanced BOPPPS teaching demonstrates consistent benefits in improving nursing students’ academic performance, self-directed learning ability and teaching satisfaction, these findings support the potential value of integrating AI into structured instructional design to enhance learning processes and student experience in nursing education.

**Systematic review registration:**

https://inplasy.com/inplasy-2026-2-0026/, Identifier: INPLASY202620026.

## 1 Introduction

The overarching goal of nursing education is to cultivate comprehensive, practice-oriented professionals with diverse competencies (1). With rapid social change and advances in medical technology, the demand for high-caliber nursing personnel has become increasingly urgent. Accordingly, exploring and implementing effective teaching approaches has emerged as a central priority in nursing education. As a discipline that requires deep integration of theory and practice, nursing curricula—such as Fundamentals of Nursing, Medical Nursing, and Surgical Nursing—not only expect students to acquire solid theoretical foundations, but also emphasize the development of clinical skills, critical thinking, and independent problem-solving abilities (2).

However, nursing courses typically involve extensive and complex content and many abstract concepts. Traditional teacher-centered lecturing often fails to foster meaningful student engagement and may contribute to a disconnect between theoretical learning and clinical practice (2).

Conventional nursing education is frequently dominated by instructors, with students positioned as passive recipients of knowledge and limited opportunities for interaction (3). This approach is not conducive to cultivating critical thinking or clinical adaptability, nor does it effectively enhance learners’ autonomy. Many students struggle to connect theoretical knowledge with clinical scenarios, resulting in difficulty adjusting to clinical placements and an inability to rapidly meet the demands of real-world practice (4).

In recent years, diversified teaching methods—such as simulation-based education and virtual reality technologies (5)—have been introduced into nursing classrooms. Nevertheless, widespread adoption is often constrained by the costs of equipment, space requirements, and faculty training, limiting scalability and broad implementation (6).

The BOPPPS teaching model is a closed-loop instructional design framework grounded in constructivist theory. It structures classroom instruction into six well-defined phases: Bridge-in, Objective, Pre-assessment, Participatory learning, Post-assessment, and Summary (5). By emphasizing goal-oriented guidance and continuous interaction, the model can improve instructional efficiency and enhance student participation. In nursing education, BOPPPS has been gradually applied across a range of courses, including Health Assessment, Nursing Management, and clinical nursing training. Existing studies suggest that, compared with traditional teaching approaches, BOPPPS demonstrates positive effects in improving academic performance, strengthening self-directed learning, and increasing teaching satisfaction (7).

However, the classical BOPPPS model still faces practical limitations in real-world implementation. For example, during the pre- and post-assessment phases, it can be difficult to provide immediate, individualized feedback for every student. Likewise, in the participatory learning phase, instructors may struggle to dynamically adjust teaching strategies based on real-time performance across an entire cohort. These challenges are particularly prominent in nursing courses characterized by diverse content and large class sizes (8).

The rise of artificial intelligence (AI) offers new opportunities to optimize the BOPPPS model. Through intelligent algorithms, learning analytics, and adaptive technologies, AI can enhance each phase of BOPPPS: enabling personalized assessment and instant feedback during pre−/post-assessments; delivering tailored learning resources and creating virtual simulation scenarios during participatory learning; and automatically generating learning diagnostic reports during the summary phase. This integrated “AI + BOPPPS” approach may support precision teaching, dynamic intervention, and a fully closed-loop evaluation process across multiple nursing courses, thereby improving overall teaching effectiveness (9).

Although some studies have begun to combine AI technologies with the BOPPPS framework and apply this enhanced model to courses such as fundamentals of nursing and medical–surgical nursing, most investigations have been small-scale and their conclusions remain inconsistent. Robust, system-level evidence is still lacking to clarify the educational benefits of an AI-enhanced BOPPPS model across the broader nursing curriculum (7).

Therefore, to comprehensively evaluate the effectiveness of the AI + BOPPPS approach across diverse nursing courses, this systematic review and meta-analysis will synthesize evidence from relevant randomized controlled trials. We will focus on the effects of this model on nursing students’ theoretical achievement, practical skills, self-directed learning ability, and teaching satisfaction, with the aim of providing high-level evidence to inform curriculum reform and innovation in nursing education.

## 2 Methods

This systematic review and meta-analysis was conducted in strict accordance with the Preferred Reporting Items for Systematic Reviews and Meta-Analyses (PRISMA) guidelines (10). PRISMA provides an evidence-based minimum set of reporting standards for systematic reviews and meta-analyses. The complete PRISMA checklist has been submitted as Supplementary material.

The study protocol was prospectively registered on the International Platform of Registered Systematic Review and Meta-analysis Protocols (INPLASY) under the registration number INPLASY202590123. The review process was performed in line with the methodological standards outlined in the Cochrane Handbook for Systematic Reviews of Interventions, to ensure rigorous quality across all stages from study design to results reporting.

### 2.1 Search strategy

We systematically searched Chinese- and English-language publications from January 2001 to October 14, 2025. The databases searched included PubMed, Web of Science, Embase, and the Cochrane Library, as well as the major Chinese databases China National Knowledge Infrastructure (CNKI), WanFang Data, and VIP (Chinese Scientific Journals Database).

The English search terms were developed by combining three main themes: the BOPPPS model, artificial intelligence, and medical education. For English-language databases, searches were performed using a combination of MeSH terms and title/abstract keywords. For Chinese-language databases, searches similarly combined subject terms with title/abstract keywords. See Supplementary material 1.

### 2.2 Inclusion and exclusion criteria

This review will include all randomized controlled trials (RCTs) that evaluate the educational effects of an artificial intelligence (AI)-enhanced BOPPPS teaching model among nursing students. Studies will be eligible if they meet the following criteria: (1) participants are nursing students; (2) the intervention involves BOPPPS integrated with AI technologies (e.g., personalized learning pathways, AI-simulated clinical cases, intelligent feedback) and is compared with traditional teaching methods or BOPPPS alone; and (3) the study reports extractable quantitative outcomes, such as theoretical examination scores, skills assessment scores, or scale-based measures of self-directed learning ability or critical thinking.

Studies will be excluded if they are not RCTs; if the intervention is not clearly described; if relevant quantitative data are not reported or the full text is unavailable; or if the publication is a duplicate.

### 2.3 Data extraction

Data extraction was performed in accordance with the Cochrane Handbook for Systematic Reviews of Interventions. Two reviewers (Huiling Zhang and Zheyuan Xia) independently screened titles and abstracts. Any disagreements were resolved through discussion, with a final decision reached in consultation with Yahui Meng.

For each included study, the following information was extracted: first author, country of origin, year of publication, sample size, intervention details (AI-enhanced BOPPPS), comparator details (control condition), and reported outcome measures.

### 2.4 Study quality assessment

All studies included in this review were randomized controlled trials. The methodological quality of the included studies was systematically assessed using the Cochrane Collaboration’s Risk of Bias tool. This instrument evaluates risk of bias across seven domains: (1) random sequence generation; (2) allocation concealment; (3) blinding of participants and personnel; (4) blinding of outcome assessors; (5) incomplete outcome data; (6) selective outcome reporting; and (7) other potential sources of bias.

Each domain was judged as having a “low risk of bias,” “high risk of bias,” or “unclear risk of bias.”

### 2.5 Statistical analysis

All statistical analyses were performed using RevMan 5.4 and Stata 17.0. Two-sided tests were applied, with the significance level set at *p* < 0.05. Pooled results are reported as effect estimates with 95% confidence intervals (95% CIs), along with heterogeneity statistics.

Because the included studies used different instruments to assess outcomes (e.g., theoretical achievement, self-directed learning ability, and teaching satisfaction), the standardized mean difference (SMD) with 95% CIs was used to synthesize continuous outcomes. A positive SMD indicates that the AI-enhanced BOPPPS teaching model was superior to the control condition (traditional teaching or BOPPPS alone).

Between-study heterogeneity was evaluated using Cochran’s Q test and the I^2^ statistic. Heterogeneity was interpreted as low when I^2^ < 25%, moderate when I^2^ = 25–75%, and high when I^2^ > 75%.

## 3 Results

### 3.1 Literature search results

We systematically searched Chinese- and English-language publications from January 2000 to October 14, 2025. The initial search identified 14 records related to the “AI-enhanced BOPPPS teaching model.” After removing duplicates in EndNote, 11 records remained. Following title and abstract screening, four records were excluded because they were not relevant to the research topic or did not involve nursing students, leaving seven studies. A supplementary search using the snowballing method yielded one additional eligible study.

**Figure 1:**
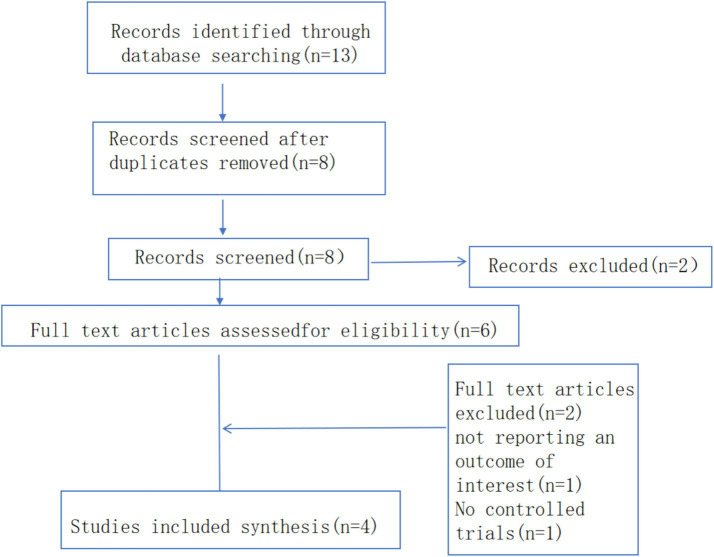
PRISMA flow diagram of study selection.

After full-text assessment of all eight articles, four were excluded due to incomplete data n=2 or because they were review articles n=2 . Ultimately, four studies met the inclusion criteria and were included in the meta-analysis (Figure 1).

### 3.2 General characteristics of the included studies

Table 1 summarizes the key characteristics of the studies included in this meta-analysis. A total of four randomized controlled trials (RCTs) published between 2000 and 2025 were included, involving 459 nursing students. Of these, 219 students were assigned to the intervention group and received the AI-enhanced BOPPPS teaching model, while 212 students in the control group were taught using traditional methods. The included trials compared educational outcomes with the “traditional teaching” group. Across the included studies, the intervention groups consistently integrated the BOPPPS teaching model with artificial intelligence (AI) technologies or digital/technology-enhanced instructional platforms. Specifically (11), implemented an AI-enhanced BOPPPS model and reported significant improvements in students’ learning adaptability, self-directed learning ability, and classroom engagement. Zhu Lina (12) applied a BOPPPS-based 5G smart classroom approach, which effectively enhanced students’ overall internship performance and teaching satisfaction. Liang Chen (13) employed a ChatGPT-assisted BOPPPS method and observed significant advantages in theoretical knowledge acquisition and clinical thinking. Hao Huijun (14) compared a BOPPPS + ChatGPT approach with traditional teaching.

**Table 1:** Characteristics of the included studies.

Author	Year	Participants (Intervention)	Participants (Control)	Comparator	Intervention	Outcomes
Yaling Zhu	2025	87	83	Traditional teaching	AI-enhanced BOPPPS teaching model	Final exam scores; classroom interaction and learning engagement; adaptive and self-directed learning ability; teaching satisfaction
Lina Zhu	2024	47	43	Traditional teaching	BOPPPS-based 5G + smart classroom	Internship performance (theory + skills + performance); self-directed learning ability; teaching satisfaction
Chen Liang	2025	58	57	Traditional teaching	ChatGPT-assisted BOPPPS teaching approach	Theoretical knowledge and clinical thinking assessment scores; classroom participation; teaching satisfaction
Huijun Hao	2025	55	29	Traditional teaching	BOPPPS + ChatGPT	Final grades; course satisfaction; perceived AI-assisted teaching; teacher–student interaction; self-directed learning; communication skills

In all included trials, control groups received conventional teacher-centered lecturing without the use of AI. Key learning outcomes assessed across studies included theoretical achievement, practical skills or internship performance, self-directed learning ability, classroom participation, learning adaptability, and teaching satisfaction. Overall, all studies reported that the AI-enhanced BOPPPS approach was significantly superior to traditional teaching across multiple dimensions (*p* < 0.05), suggesting that this model is effective and has strong potential for wider adoption in nursing education.

### 3.3 Risk of bias assessment of included studies

The quality assessment indicated that the included studies had an overall low risk of bias and generally good methodological quality. All trials reported complete outcome data, and no evidence of selective outcome reporting was identified.

Regarding random sequence generation, all four studies reported using random allocation; however, some did not clearly describe the specific randomization procedures and were therefore rated as having an unclear risk of bias. For allocation concealment, most studies did not provide sufficient methodological details and were likewise judged as unclear risk.

With respect to blinding of participants and personnel, the studies by Hao Huijun (14) and Zhu Lina (11) did not adequately report or implement blinding, resulting in a high risk of performance bias. In contrast, blinding of outcome assessors was well controlled in most studies and was assessed as low risk.

For incomplete outcome data, all four studies reported complete follow-up and outcome data, with no apparent loss to follow-up or withdrawals; therefore, this domain was judged as low risk. No concerns related to selective reporting or other sources of bias were identified, and these domains were also rated as low risk.

**Figure 2:**
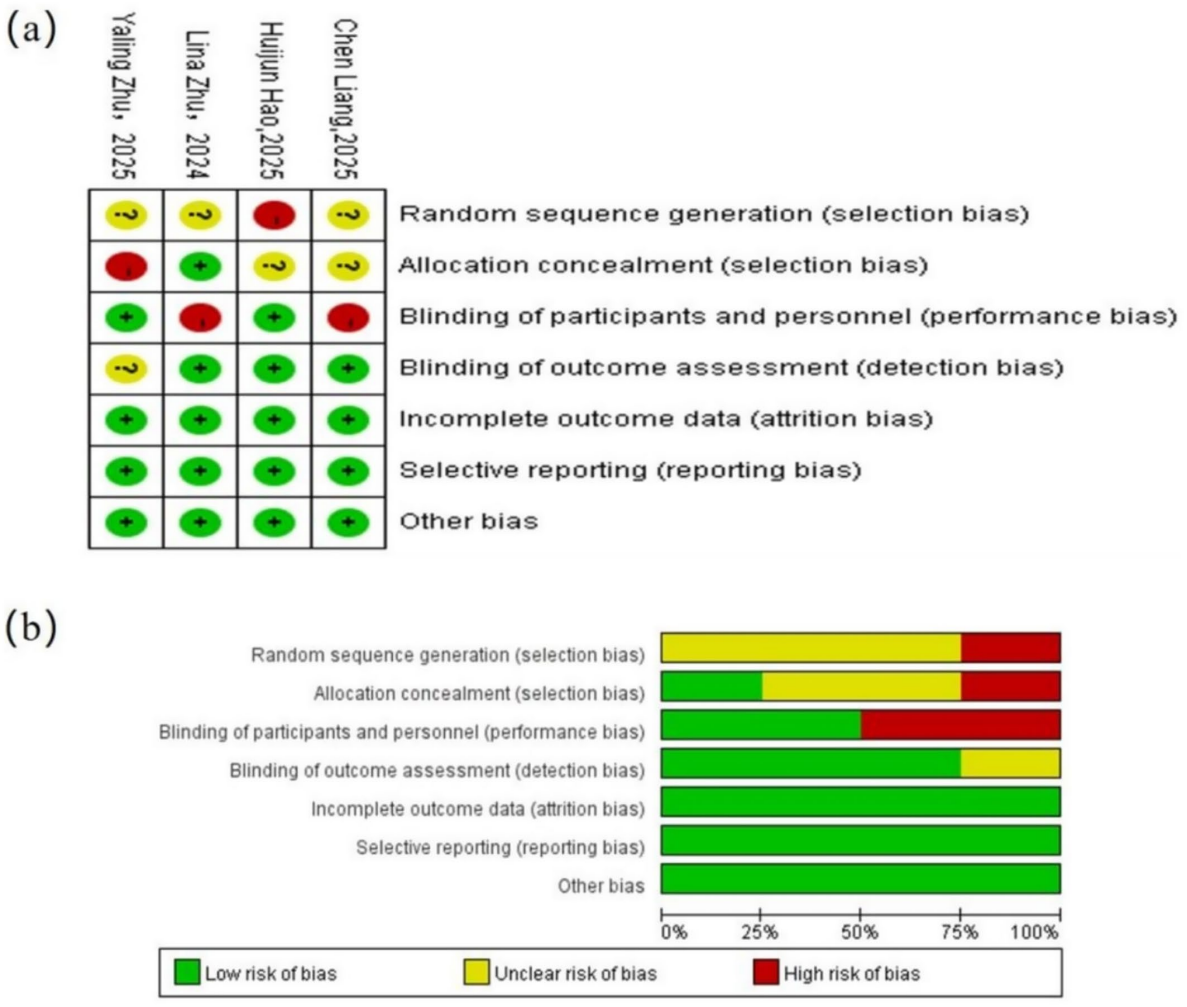
Risk-of-bias assessment of included studies: **(a)** Risk-of-bias summary; **(b)** risk-of-bias graph.

In summary, the four included RCTs were of relatively high quality overall, with the main potential risks of bias arising from insufficient reporting of randomization procedures and challenges in blinding. The visual summary of the risk-of-bias assessment is presented in Figure 2.

### 3.4 Results

A total of four randomized controlled trials (RCTs) were included in this study, evaluating the effects of an AI-enhanced BOPPPS teaching model on nursing students’ theoretical achievement, self-directed learning ability, and teaching satisfaction. The total sample size was 459 participants, including 219 in the intervention group and 212 in the control group.

#### 3.4.1 Academic performance

**Figure 3:**
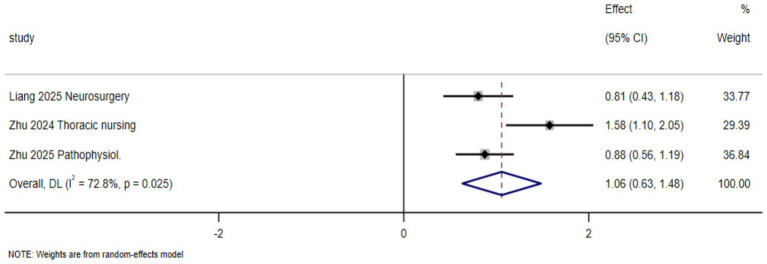
Effects of the AI-enhanced BOPPPS teaching model on academic performance in nursing students.

Three studies reported students’ theoretical or overall academic performance. The meta-analysis showed a statistically significant improvement in academic performance in the AI-enhanced BOPPPS group compared with the control group (SMD = 1.06, 95% CI: 0.63–1.48; *p* = 0.025). The heterogeneity among studies was substantial (I^2^ = 72.8%), indicating variability across studies (Figure 3).

#### 3.4.2 Self-directed learning ability

**Figure 4:**
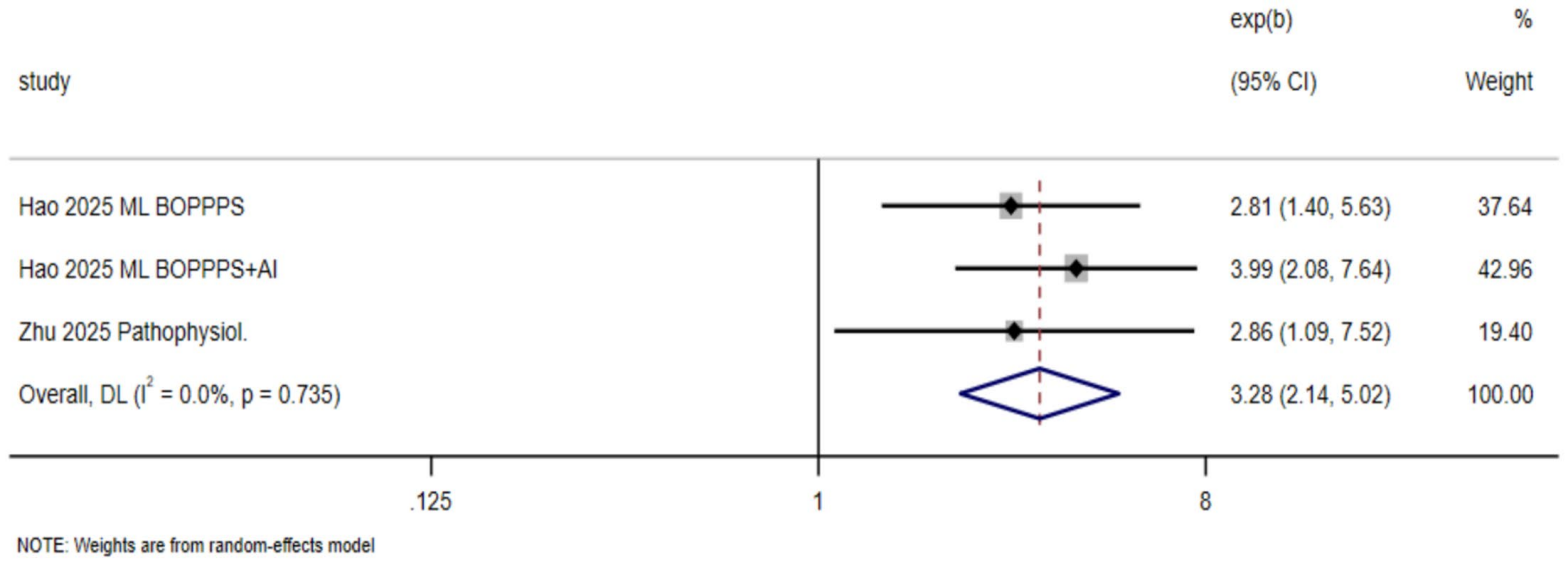
Effects of the AI-enhanced BOPPPS teaching model on self-directed learning ability in nursing students.

Three studies reported outcomes related to self-directed learning ability, comparing BOPPPS, BOPPPS + AI, and traditional teaching approaches. The meta-analysis showed that the proportion of students rated as “excellent” in self-directed learning ability was significantly higher in the intervention group than in the control group (RR = 3.28, 95% CI: 2.14–5.02; *p* < 0.001), indicating that the AI-enhanced BOPPPS model substantially improved students’ self-directed learning ability. Heterogeneity was negligible I^2^ =0.0%,p=0.735 , suggesting high between-study consistency and a robust pooled estimate (Figure 4).

#### 3.4.3 Teaching satisfaction

**Figure 5:**
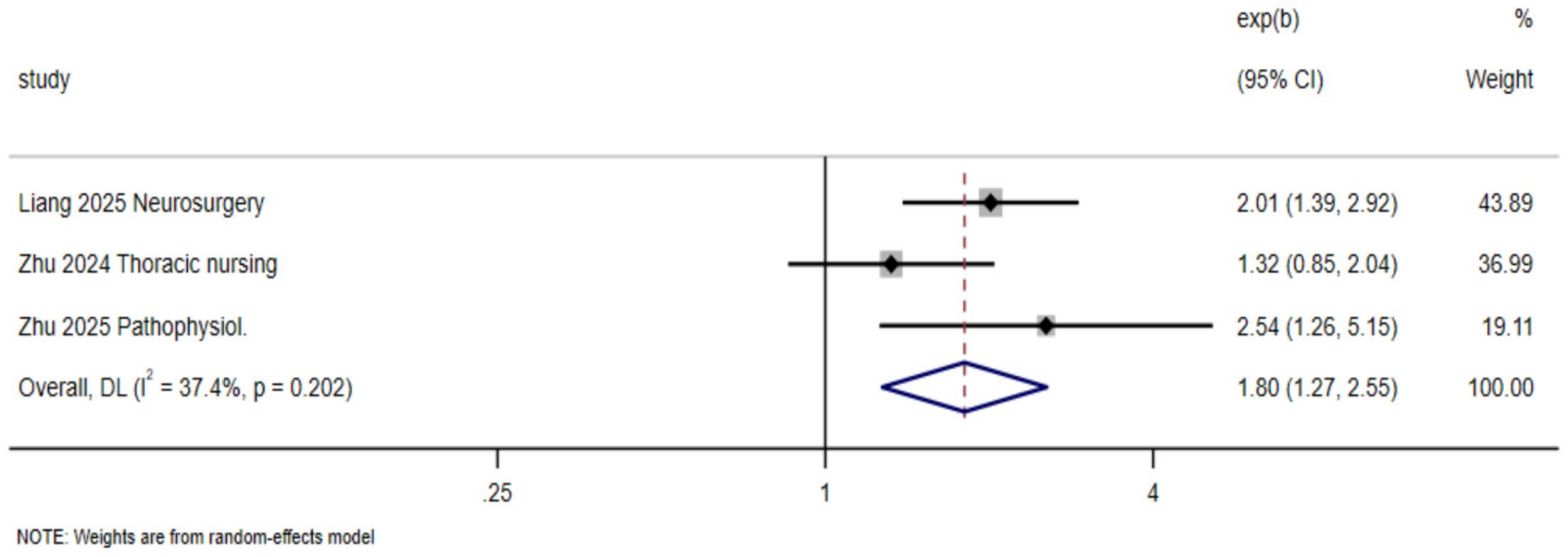
Effects of the AI-enhanced BOPPPS teaching model on teaching satisfaction in nursing students.

Three studies assessed students’ satisfaction with the teaching approach, with “very satisfied” defined as the event of interest. The meta-analysis showed that the AI-enhanced BOPPPS model significantly increased teaching satisfaction compared with the control condition (RR = 1.80, 95% CI: 1.27–2.55; *p* < 0.001), indicating a substantial improvement. Between-study heterogeneity was low to moderate I^2^ =37.4%,p=0.202 (Figure 5).

## 4 Discussion

This study systematically synthesized evidence from randomized controlled trials that have integrated artificial intelligence (AI) technologies into the BOPPPS teaching model in recent years, with the aim of evaluating its effectiveness in nursing education. Overall, although the AI-enhanced BOPPPS model showed a significant positive effect on academic performance (SMD = 1.06, 95% CI: 0.63–1.48, *p* = 0.025), there was substantial heterogeneity among the studies (I^2^ = 72.8%). The variability in outcomes may be due to differences in AI implementation, course types, assessment formats, and student baseline characteristics. These factors may have influenced the degree of improvement observed in different studies. It produced stable and significant benefits in enhancing students’ self-directed learning ability and teaching satisfaction. These findings suggest that the value of the AI-enhanced BOPPPS innovation may lie less in short-term, test-based indicators of knowledge acquisition and more in deeper changes in the learning process, learning behaviors, and learning experience. This aligns with the international shift in nursing education from a focus on “knowledge transmission” toward greater emphasis on learning processes and competency development (15).

The studies included in the meta-analysis varied in terms of AI implementation, course types, and assessment formats. To provide more context, specific AI tools played a significant role in driving the observed positive outcomes in the studies. AI-based question-answering systems were used in several studies to provide personalized feedback and assist students in addressing their knowledge gaps in real-time. By offering instant answers to students’ questions, these systems enhanced self-directed learning and supported deeper engagement with the material. Additionally, several studies implemented AI-powered adaptive learning platforms that personalized content delivery based on individual learning progress. This helped to optimize learning efficiency and engagement, fostering better understanding and retention of key concepts. Some studies also incorporated AI-driven simulation tools, which allowed students to engage in practical, scenario-based learning. This significantly improved clinical reasoning, problem-solving skills, and decision-making abilities. These AI tools collectively contributed to the overall positive impact on academic performance, but the variation in their usage and implementation may explain the heterogeneity observed across studies.

The introduction of AI provides substantial added value across several key stages of the BOPPPS model. Theoretically, BOPPPS is a constructivist-oriented, structured instructional framework that promotes students’ active construction of meaning through clearly defined objectives, ongoing feedback, and participatory learning. When AI technologies are embedded within this structure, they can markedly strengthen personalized feedback—an element that is often difficult to deliver in traditional classrooms due to time constraints and high student–teacher ratios (16). AI can generate feedback reports based on students’ real-time learning behaviors, recommend adaptive learning resources, and dynamically track individual progress during the pre- and post-assessment phases, thereby forming a “technology-driven closed loop” of formative assessment (17).

Such continuous support enhances students’ sense of control over learning and perceived learning efficacy, which in turn facilitates the development of self-directed learning ability. This mechanism closely aligns with the core processes described in Self-Regulated Learning Theory, suggesting that the AI-enhanced BOPPPS approach not only changes how students learn but may also reshape their learning strategies and motivation (18).

In addition, from the perspective of Cognitive Load Theory, nursing curricula are characterized by complex content and dense knowledge structures. Students often struggle to build deep understanding because of a high level of extraneous cognitive load. AI-supported structured content delivery, real-time explanations, and simulated learning scenarios may substantially reduce extraneous load, allowing learners to allocate more cognitive resources to the essential learning tasks (19). This may help explain why students consistently reported higher classroom engagement, autonomy, and overall learning experience. The marked improvement in satisfaction further supports the notion that technology-enhanced instruction can positively influence learners’ emotional experience, perceived value of learning, and professional identity.

In this meta-analysis, the pooled effect on academic performance was not stable and exhibited substantial heterogeneity. This finding does not necessarily indicate that the AI-enhanced BOPPPS model is ineffective in promoting theoretical knowledge; rather, it likely reflects the influence of multiple interacting factors. First, the included studies varied considerably in how AI was implemented—ranging from intelligent question-answering systems and smart classroom platforms to adaptive learning resources and AI-generated cases—resulting in differences in instructional functions and depth. Second, variations in course types, assessment formats, difficulty levels, and scoring criteria inevitably contributed to variability in achievement outcomes. Moreover, written or theoretical examination scores may not adequately capture higher-order competencies such as clinical reasoning, problem-solving, or the ability to transfer knowledge across contexts, potentially underestimating AI’s contribution to deeper learning (20).

As nursing education increasingly moves away from a score-centered evaluation paradigm toward competency-based education (CBE) (21), future research should develop and adopt more comprehensive outcome measures that better reflect professional competence. Such measures would enable a more accurate and holistic assessment of the true educational impact of AI-enhanced teaching models.

## 5 Limitations

Although the findings of this study offer meaningful insights, the current evidence should be interpreted with caution due to several limitations. First, the number of included studies was small, and most were single-center trials, which may limit the generalizability of the results. Second, there is no standardized approach to AI integration across studies; differences in the intensity and depth of the intervention, instructors’ technical proficiency, and classroom contexts may have contributed to variability in outcomes. Third, most studies evaluated only short-term interventions and lacked follow-up on long-term learning trajectories, clinical internship performance, critical thinking, and clinical competence—outcomes that are central to nursing education. In addition, blinding was inadequately implemented in some trials, which may have introduced observer bias. These issues highlight the need for more rigorous study designs in future research.

From a broader educational perspective, integrating AI with structured instructional design is not merely a technological add-on, but may represent a reconfiguration of the nursing education ecosystem. Through real-time feedback, adaptive resource delivery, and scalable learning materials, AI can function as a continuous learning companion, enabling students to shift from passive recipients to active managers of their learning processes. Meanwhile, instructors’ roles may evolve from knowledge transmitters to learning facilitators, interpreters of learning analytics, and designers of learning experiences. This transformation aligns closely with the learner-centered orientation increasingly emphasized in nursing education and may foster stronger clinical adaptability, problem-solving capacity, and lifelong learning skills—capabilities essential for navigating the complexity of future healthcare environments.

## 6 Conclusion

This systematic review and meta-analysis indicates that integrating artificial intelligence (AI) technologies into the BOPPPS teaching model can significantly enhance nursing students’ self-directed learning ability and learning satisfaction, highlighting AI’s unique value in optimizing learning processes, strengthening motivation, and improving classroom experiences. Although the pooled effect on academic performance was not consistently significant, this inconsistency likely reflects variations in course structures, assessment methods, and the depth of AI integration rather than a lack of efficacy of the model itself. Overall, the AI-enhanced BOPPPS model showed a significant positive effect on academic performance, although the variability in the studies suggests the need for further research with more consistent implementation and larger sample sizes.

Future research should further standardize the core components of AI-supported teaching interventions, develop more comprehensive evaluation frameworks that better capture nursing competence, and employ multicenter studies with long-term follow-up to verify potential benefits in critical thinking, clinical reasoning, and clinical competency development. As intelligent technologies continue to converge with educational theory, AI-enabled structured teaching models are poised to become an important direction for innovation in nursing education, offering new pathways to cultivate high-quality nursing professionals capable of thriving in increasingly complex healthcare environments.

## Data Availability

The original contributions presented in the study are included in the article/Supplementary material, further inquiries can be directed to the corresponding author.
