# Supplementary material for: Effects of an AI-enhanced BOPPPS teaching model in nursing courses: a meta-analysis of randomized controlled trials
Source: Front Med (Lausanne). 2026 Feb 18;13:1767911. doi: 10.3389/fmed.2026.1767911 (PMC12956507; doi:10.3389/fmed.2026.1767911)
Supplement: Supplementary file 2 [file Table_1.docx]

**Supplementary Table 1**

**PubMed search strategy**

| **Step** | **Search terms (PubMed)** |
| --- | --- |
| #1 | BOPPPS[tiab] OR "BOPPPS model"[tiab] OR "BOPPPS teaching"[tiab] OR "Bridge-in"[tiab] |
| #2 | "Artificial Intelligence"[mh] OR "Machine Learning"[mh] OR "Natural Language Processing"[mh] OR "artificial intelligence"[tiab] OR AI[tiab] OR "machine learning"[tiab] OR "deep learning"[tiab] OR "generative ai"[tiab] OR "large language model*"[tiab] OR LLM*[tiab] OR ChatGPT[tiab] OR GPT[tiab] OR "intelligent tutoring system*"[tiab] OR "adaptive learning"[tiab] OR "smart classroom"[tiab] OR 5G[tiab] |
| #3 | "Education, Nursing"[mh] OR "Students, Nursing"[mh] OR nursing[tiab] OR nurse*[tiab] OR "nursing education"[tiab] OR "nursing student*"[tiab] OR "nursing course*"[tiab] |
| #4 | "randomized controlled trial"[pt] OR "controlled clinical trial"[pt] OR randomi?ed[tiab] OR randomly[tiab] OR RCT[tiab] OR trial[tiab] NOT (animals[mh] NOT humans[mh]) |
| #5 | #1 AND #2 AND #3 AND #4 |
| #6 (Final) | (#1 AND #2 AND #3 AND #4) AND ("2001/01/01"[dp] : "2025/10/14"[dp]) AND (english[lang] OR chinese[lang]) AND humans[mh] |
